# Geometric parameters that affect the behavior of logic-gated CAR T cells

**DOI:** 10.3389/fimmu.2024.1304765

**Published:** 2024-01-26

**Authors:** Alexander C. Partin, Richele Bruno, Sanam Shafaattalab, Erica Vander Mause, Aaron Winters, Mark Daris, Casey Gahrs, Claudia A. Jette, Breanna DiAndreth, Mark L. Sandberg, Agnes E. Hamburger, Alexander Kamb, Timothy P. Riley

**Affiliations:** A2 Biotherapeutics, Inc., Agoura Hills, CA, United States

**Keywords:** logic-gate, CAR (chimeric antigen receptor), synapse, T cell, immunotherapy

## Abstract

Clinical applications of CAR-T cells are limited by the scarcity of tumor-specific targets and are often afflicted with the same on-target/off-tumor toxicities that plague other cancer treatments. A new promising strategy to enforce tumor selectivity is the use of logic-gated, two-receptor systems. One well-described application is termed Tmod™, which originally utilized a blocking inhibitory receptor directed towards HLA-I target antigens to create a protective NOT gate. Here we show that the function of Tmod blockers targeting non-HLA-I antigens is dependent on the height of the blocker antigen and is generally compatible with small, membrane-proximal targets. We compensate for this apparent limitation by incorporating modular hinge units to artificially extend or retract the ligand-binding domains relative to the effector cell surface, thereby modulating Tmod activator and blocker function. By accounting for structural differences between activator and blocker targets, we developed a set of simple geometric parameters for Tmod receptor design that enables targeting of blocker antigens beyond HLA-I, thereby broadening the applications of logic-gated cell therapies.

## Introduction

Engineered immune cells have emerged as a powerful platform to reprogram immunological function, and redirecting T cells to target and kill tumor cells has become a therapeutic strategy of significant interest. Several modalities aim to accomplish this goal (1), and replacing or supplementing the existing T cell receptor (TCR) with alternative receptors to obtain the desired specificity has become commonplace (2).

Chimeric antigen receptors (CARs) have been particularly effective at redirecting T cells to eradicate B cell malignancies, and efforts to expand this approach into solid tumor indications are ongoing (3). However, unlike TCRs, CARs only have access to the extracellular proteome, greatly restricting the available targets. Thus, a key obstacle in oncology is a lack of targets that specifically differentiate tumors from normal tissues. Several approaches (4) have circumvented this challenge by implementing logic-gated systems for the detection and response to combinatorial antigen profiles, rather than single tumor-associated antigens (5, 6). One such example incorporates the use of synthetic Notch (synNotch) receptors that release activating or inhibitory transcription factors upon antigen recognition (7). Alternatively, the Tmod™ platform pairs an activating CAR or TCR (i.e., activator module) with an engineered inhibitory receptor (i.e., blocker module) to provide a safety switch to spare normal cells that express an inhibitory antigen not typically found in tumor cells. A compelling opportunity to target tumors selectively with Tmod constructs involves patients whose tumors have clonal loss of heterozygosity (LOH) at the Human Leukocyte Antigen (HLA) locus, a phenomenon observed in ~15% of cancers (4). In this circumstance, tumor selectivity is controlled by a specific HLA allele expressed on normal tissues but absent in tumor cells.

In principle, activators and blockers are modular and can accommodate many different ligand-binding domains (LBDs (8)). The modularity of these engineered receptors provides the flexibility to target a broad suite of unique antigens, addressing the need for new cancer targets. CAR design has evolved over the last few decades (9) and the widely accepted 3^rd^ generation platform used here is typically composed of intracellular signaling domains from CD28, 4-1BB, and CD3ζ fused to an extracellular scFv to impart antigen regulation. The blocker developed as part of the Tmod system has a similar modular design, and leverages components from the LIR-1 protein (encoded by the gene leukocyte immunoglobulin-like receptor subfamily B member 1, *LILRB1*), a receptor expressed in specific cells of the immune system (10). By coupling the LIR-1 transmembrane domain (TM) and intracellular domain (ICD) to an LBD specific to antigens expressed on healthy tissues, the blocker enforces tumor selectivity of the activating CAR (8).

Despite the simplicity of CAR design, the detailed mechanisms that underlie CAR function remain elusive (11). Manipulating parameters such as affinity, avidity, and signaling domain composition can often improve CAR-T function, although no single feature appears to be dominant (12). Target selection also has a dramatic effect when manipulating T cell behavior, as both CARs and bispecific T cell engagers (13, 14) tend to be less potent when targeting large and bulky antigens (15). These observations mirror the kinetic segregation model of T cell activation (16), where the relatively narrow intercellular distances achieved with a TCR:pMHC interaction is fundamental to T cell activation (17). Recent experiments with CARs explore these design principles using spacer domains that connect the LBD to the transmembrane domain to tune the intercellular distance between T cell and target cell (18–20).

**Figure 1:**
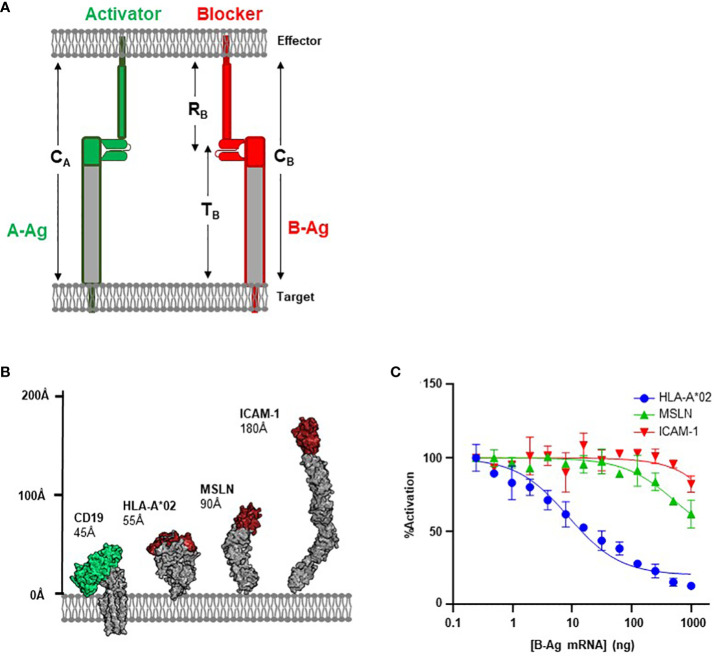
Antigen Height as a Functional Parameter. **(A)** Schematic of structural parameters in Tmod platform. See **Table 1** for parameter definitions. **(B)** Structural comparison of CD19 A-Antigen (green) and three B-Antigens (HLA-A*02, MSLN, and ICAM-1; red). **(C)** Jurkat T cells expressing the CD19 activator and a blocker targeting one of the three B-antigens described above co-cultured with K562(CD19+) target cells transfected with increasing amount of B-antigen mRNA.

Here, we apply similar concepts to the component receptors of the Tmod system. Although the Tmod platform has demonstrated preclinical success targeting HLA-I antigens with the blocker (21), expanding Tmod to a broader patient population will require the design of new blocker modules tailored to other antigens. Considering the diversity of the human proteome regarding protein size and structure, selecting blockers with optimal function to expand the Tmod platform represents a significant challenge. We conceptualized a series of geometric parameters that influence Tmod behavior (**Figure 1A**). Our data demonstrate that ideal blocker targets share similar size constraints as the activator antigen, but rational receptor engineering can restore function with suboptimal targets. Surprisingly, lengthening the activating and/or inhibitory receptor can restore function of suboptimal Tmod antigen pairs, providing a simple solution to the challenge of controlling functional selectivity with large target antigens. This work provides a blueprint for Tmod design, captured in a set of simple geometric rules that expands the set of potential targets for cell therapy.

## Methods

### Structural modeling

Antigens were modeled and measured in Pymol using coordinates derived from PDB IDs 7JIC (CD19), 6AMT (HLA-A*02), or 1Z8L (PSMA). For antigens without a complete experimental structure (CEA, ICAM1, MSLN), starting coordinates were derived from Alphafold (22). All structures were relaxed and re-modeled using Rosetta with the ref2015 score function and the Pyrosetta interface (23).

### Construct design and cloning

scFvs were designed using flexible (G4S)3-GG linker to connect the VH and VL domains. All third-generation activator CAR constructs contained the CD8a or EGF-like hinges fused to CD28 TM, as well as CD28, 4-1BB, and CD3z ICDs. All blocker receptor constructs contained either the LIR-1 or EGF-like hinges fused to the LIR-1 TM and ICD. Templates used for target protein mRNA synthesis contained 5′ T7 promoter followed by the V kappa 1 signal peptide and codons encoding either HLA, MSLN, ICAM-1, CEA, or PSMA. For FLAG-based blocking assays, a FLAG sequence (DYKDDDDK) was inserted N-terminally to the mature protein sequence. All DNA constructs were assembled using Golden Gate Assembly. DNA templates were either amplified by PCR or linearized by restriction enzyme digest, then mRNA was synthesized using the HiScribe T7 ARCA mRNA kit (New England Biolabs). The *in vitro* synthesized mRNA was purified using the Monarch RNA Cleanup Kit (New England Biolabs), eluted in 1 mM sodium acetate, and stored at −80°C.

### Surface plasmon resonance binding data and analysis

Surface plasmon resonance experiments were performed with a Biacore 3000 instrument using CM5 sensor chips. In all experiments, recombinantly expressed scFvs were immobilized on the sensor chip via standard amine coupling and monovalent antigens were injected as analyte. Experiments were performed at 25°C in 20mM HEPES (pH7.4), 150mM NaCl, and 0.005% surfactant P-20. Injected analyte concentrations spanned 1-1000nM. Data were processed using BiaEvaluation 4.

### Target cell mRNA transfection

On the day of transfection, target cells (HeLa or K562, depending on experiment) were counted, washed with 1x PBS and resuspended to 1.1e7vc/mL in SE (for HeLa cells) or SF (for K562 cells) transfection buffer (Lonza). Target antigen mRNA was serially diluted 2-fold across 15 points in SE or SF transfection buffer in 96-well v-bottom plates. Target cells were added to each well containing the mRNA at a concentration of 1.33e7vc/mL. The mRNA/cell mixture was transferred to a 16-well Lonza 4D cuvette and electroporated according to the manufacturer’s protocol established for target cell line. Post-transfection, the cells were immediately placed into MEM growth media containing serum and seeded into rows of 384-well culture plates at a density of 5,000–10,000cells/well, depending on experiment. Remaining transfected cells were seeded into separate 96-well plates for expression testing by flow cytometry. Plates were cultured for >16 hours at 37°C and 5% CO2.

### Jurkat co-culture assay

Jurkat-NFAT luciferase cells were counted, washed with 1x PBS and 2e6 viable cells were resuspended in 120ml of R2 buffer (ThermoFisher Scientific) containing 4-8 μg of a 1:1 DNA mixture encoding appropriate activator and blocker receptor constructs. Cells were transfected with the Neon Transfection System (Invitrogen) using the 100 µl format with E2 Buffer (1500 Volts, 10 width, 3 pulses). Cells were cultured overnight at 37°C, 5% CO2 in RPMI containing 20% FBS for co-culture assays the following day. 5,000-10,000 activating and blocker receptor expressing Jurkat-NFAT luciferase cells were combined with 5,000-10,000 transfected target cells expressing a fixed amount of activator antigen as described above in triplicate wells of a 96-well plate. The co-culture plates were incubated at 37°C, 5% CO2 for 6 hours. Twenty microliters of luciferase substrate (BPS Biosciences) was added to each well of the plate. The plate was then incubated at room temp for 15 minutes and read on a Tecan M1000 luminescent plate reader with 100ms integration time/well. Percent inhibition was interpreted as the ratio between luminescence from Jurkat cells co-cultured with target cells treated with the highest mRNA concentration of blocker target, and luminescence from Jurkat cells co-cultured with target cells expressing no blocker target.

### Primary T killing assay

Primary human T cells were transduced with two lentiviral constructs encoding the CD19 CAR and MSLN blocker. Cells were grown in G-Rex plates according to the manufacturer’s instructions in X-VIVO 15 media (Lonza Bioscience) supplemented with 1% human serum and 300 IU/mL IL-2. After 10 days, samples were taken from each well, counted, and stained with recombinant CD19 and MSLN to estimate transduction efficiency. Transduced cells were initially enriched using biotinylated MSLN, and subsequent cell enrichment was performed with recombinant MSLN to remove activator-only cells.

Renilla luciferase (Rluc)(+) RFP(+) Raji target cells were used to enable visualization by ImageXpress Micro (Molecular Devices) and terminal luminescence measurements. Rluc(+) RFP(+) Raji targets were co-cultured with Tmod T cells at effective E:T ratios ranging from 0.001-2.7 (0.01-27 actual E:T) for 48 hours. Using Renilla luciferase substrate (Promega), relative luminescence values were captured, and a specific killing percentage was calculated. MetaXpress software was used to calculate RFP(+) surface area between conditions and verify T cell killing (Data not shown).

### Flow cytometry

Approximately 100,000 cells were collected from each transfected group for flow cytometric analysis. The cells were washed three times in cold PBS containing 1% BSA, then incubated with the corresponding primary antibody for 45 minutes on ice using the manufacturers’ recommended staining concentrations. To prepare antigen probes, biotinylated proteins were incubated with PE-conjugated streptavidin at a 4:1 ratio for 20 minutes at room temperature, and quenched using RPMI media containing 2% v/v FBS and diluted to 10 μg/mL using PBS/BSA. The cells were then washed 3 times in PBS/BSA and incubated with secondary antibody (see below) using the manufacturers’ recommended staining concentrations. Cells were then loaded for flow cytometry data acquisition using a BD FACSCanto (BD Biosciences) and FACSDiva software (BD Biosciences). Forward scatter (FSC) *vs* side scatter (SSC) measurements were used to gate for single cells. Flow cytometric analysis was performed using FlowJo software.

Quantitative analysis of antibody-binding capacity was performed using the QIFIKIT (Dako) according to manufacturer’s instructions. Briefly, during flow cytometry sample preparation, 50 μL of resuspended QIFI bead slurry was incubated with secondary antibody using the same protocol as the sample cells. After 3 washes in PBS/BSA, the QIFI beads were loaded on the FACS Canto, using the same parameters as the sample cells being interrogated. Using FlowJo, median fluorescence intensity (MFI) was calculated for each of 6 species of QIFI beads containing different densities of antibody-binding sites, then converted into antibody-binding capacity using values provided by the manufacturer. A calibration curve was generated from these values, which was then used to convert sample cell MFI values into antibody-binding capacities.

Antibodies used in this study: Mouse anti-huHLA-A*02 (BD Biosciences, Cat# 551230); F(ab’)2-Goat anti-Mouse IgG (H+L) cross-adsorbed secondary antibody, Alexa Fluor 647 (Invitrogen, Cat# A21237); Mouse anti-huMSLN (R&D Systems, Cat# MAB32653); Mouse anti-huICAM-1 (R&D Systems, Cat# MAB720); Mouse anti-FMC63, PE (Acro Biosystems, Cat#FM3-HPY53); biotinylated human mesothelin (296-580) protein, His, Avitag (Acro Biosystems, Cat#MSN-H82E9); PE Streptavidin (BioLegend, Cat# 405245).

## Results

### Section 1: height of the B-antigen influences blocking

CAR-T platforms have generally been more successful when targeting small proteins over large and bulky antigens (15, 24). We hypothesized that the same principles would apply to the inhibitory blocker module used for Tmod; i.e., smaller blocker antigens (B-antigens) would translate to improved inhibitory responses. The first iterations of the Tmod blocker were developed to target the relatively small (55Å axial length; **Figure 1B**) extracellular domain of HLA-A*02, which can trigger exceptional inhibitory behavior (8, 21). However, expansion into larger patient populations independent of HLA LOH status requires the ability to engage non-HLA targets (25).

To investigate the effectiveness of a blocker module reprogrammed to target B-antigens of different heights, we compared blockers directed at two non-HLA antigens with the established HLA-A*02 blocker (8). We used an established CD19 CAR with the FMC63 scFv in a 3^rd^-generation backbone as the activator (26, 27), paired with three different blocker constructs targeting membrane-distal epitopes of three distinct B-antigens (HLA-A*02, MSLN, and ICAM-1; **Figure 1B**). The affinities of the scFvs for their cognate B-antigens ranged from 30 to 200 nM (**Supplementary 1**). These model B-antigens cover a range of ~100Å axial lengths and were intended to explore the modularity of the Tmod platform regarding B-antigen height.

We measured the effectiveness of each blocker by evaluating the ligand-dependent inhibition of the CD19-mediated CAR activation. The genes for each receptor combination were co-transfected into Jurkat cells expressing an NFAT-luciferase reporter. Jurkat cells were co-cultured with target cell lines expressing a fixed level of CD19 and increasing levels of B-antigen (generated by transfection with increasing amounts of B-antigen synthetic mRNA) to generate dose-inhibition curves. We characterized the level of inhibition with two parameters: IC_50_ (B-antigen concentration at half-maximal inhibition) and I_max_ (maximum percentage of inhibition).

As expected, the blocker targeting the HLA-A*02 B-antigen efficiently inhibited Jurkat cell activation in a concentration-dependent manner. Indeed, at the highest HLA-A*02 mRNA concentration, this blocker module inhibited approximately 90% of the CD19 CAR activation signal (**Figure 1C**). In contrast, blockers targeting MSLN and ICAM-1 were less effective at inhibiting the CD19 CAR (40% and 20%, respectively). The relative amount of inhibition was anti-correlated with the height of the blocker target, with the small HLA-A*02 (55Å) outperforming both the larger MSLN (90Å) and ICAM-1 (185Å). As other basic parameters of CAR function (e.g., affinity and expression; **Supplementary 1**) do not explain the decrease in blocking function, these data suggests that the larger blocker targets contribute to an overall receptor/ligand complex height (combined height or C_B_; **Figure 1A**; **Table 1**) that compromises Tmod function.

**Table 1:** List of parameters influencing tmod function.

R_A_ = Height of CAR extracellular region from effector cell membrane R_B_ = Height of Blocker extracellular region from effector cell membrane T_A_ = Total height of A-Antigen extracellular region from target cell membrane T_B_ = Total height of B-Antigen extracellular region from target cell membrane E_A_ = A-Antigen epitope distance from target cell membrane E_B_ = B-Antigen epitope distance from target cell membrane X_A_ = Length of A-Antigen distal from activator epitope (T_A_ – E_A_) X_B_ = Length of B-Antigen distal from blocker epitope (T_B_ – E_A_) C_A_ = Combined intermembrane distance spanned by Activator/A-Antigen complex (R_A_ + E_A_) C_A_ = Combined intermembrane distance spanned by Blocker/B-Antigen complex (R_A_ + E_A_)

### Section 2: targeting membrane-proximal epitopes results in more effective inhibition

**Figure 2:**
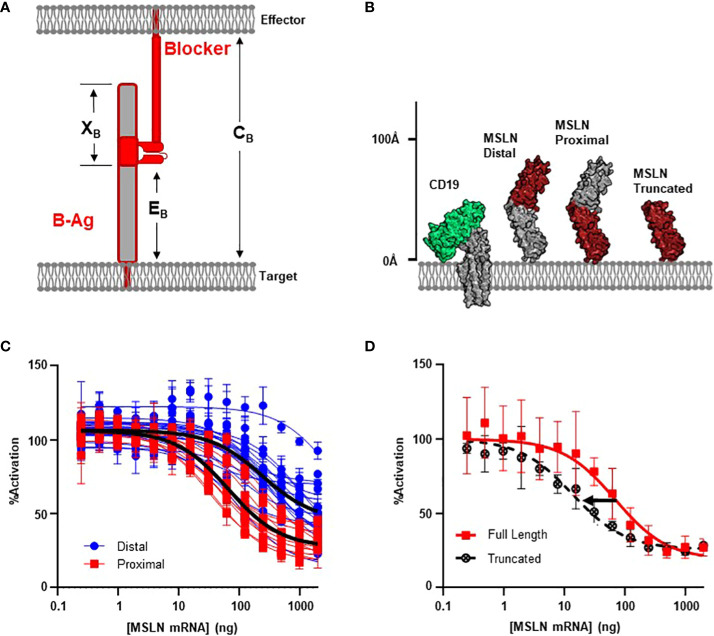
MSLN Blocker Behavior is Influenced by Epitope. **(A)** Selection of target epitope is a potential parameter that can influence C_B_. See **Table 1** for parameter definitions. **(B)** MSLN epitopes are either membrane distal or membrane proximal. Binders recognizing proximal epitopes can also recognize a truncated form of MSLN that is approximately the same height as CD19. **(C)** Jurkat T cells expressing the CD19 activator and a blocker targeting either a MSLN-distal epitope (N=17) or a MSLN-proximal epitope (N=11) co-cultured with K562(CD19+) target cells transfected with increasing amount of MSLN mRNA. Average trace for each epitope bin shown in black line (p< 0.001). **(D)** Jurkat T cells expressing the CD19 activator and a membrane proximal MSLN blocker co-cultured with K562(CD19+) target cells transfected with mRNA encoding either the full-length or truncated form of MSLN mRNA.

The sharp decrease in blocking function associated with larger B-antigens implies that there is an upper limit to combined height (C_B_) for an effective blocker. This is analogous to the reported restrictions on the activation synapse (C_A_) observed with CARs and TCRs (28), although antigen size is not the sole predictor of T cell antigen response (12, 29). One strategy shown to improve T cell antigen sensitivity is the deliberate targeting of membrane-proximal epitopes (15, 30). Therefore, we considered how the proximity of the B-antigen epitope (E_B_) relative to the target cell membrane may also influence blocking function, presumably by altering C_B_ (**Figure 2A**). However, targeting membrane-proximal epitopes of long or bulky antigens may also limit epitope accessibility, and therefore receptor engagement, by creating the potential for steric interference of the B-antigen distal region with the effector cell membrane. We thus also consider the length of the epitope distal region on the blocker antigen (X_B_).

After comparing the length of ICAM-1 to native TCR:pMHC complexes (**Figure 1B**; **Supplementary 2**), we noted that the high X_B_ values of membrane-proximal ICAM binders may introduce a steric clash with the effector cell membrane. Therefore, we selected the mid-sized MSLN B-antigen from above to explore these variables. We screened 36 anti-MSLN LBDs with the intention to diversify E_B_ (31) and used MSLN truncations to functionally define and classify the membrane-distal and membrane-proximal binders (**Supplementary 3**). After conversion into the blocker format, we assessed blocking potencies by the Jurkat activation assay described above. Clustering anti-MSLN blockers by epitope revealed that blockers targeting membrane-proximal MSLN epitopes were generally more effective at blocking the CAR activation signal compared to those targeting membrane-distal epitopes (p< 0.001, **Figures 2B, C**). On average, blockers generated from membrane-proximal MSLN binders inhibited over 70% of the maximum activation signal when co-cultured with CD19(+) target cells transfected with MSLN mRNA. This contrasted with the reduced potencies of blockers derived from membrane-distal MSLN binders (I_max_ ~70% *vs* 50% inhibition; IC_50_ ~60 ng *vs* 250 ng MSLN mRNA) (**Figure 2C**). Although blocker function in these experiments was not normalized for expression level and affinity, the data here imply that the inhibitory mechanism of the blocker has a significant dependence on the epitope distance from the membrane.

As mentioned above, targeting membrane-proximal epitopes of large antigens must consider the extracellular antigen bulk beyond the epitope. Indeed, the full-length MSLN protein extends ~50% further from the target cell membrane than HLA-A*02 (**Figure 1B**), and this additional mass of extracellular domain (X_B_, **Figure 2A**) may interfere with blocking, regardless of epitope position. To directly interrogate the effect of the excess MSLN extracellular domain (X_B_), we selected the most sensitive MSLN blocker from above and repeated the inhibition experiment with a truncated form of MSLN (**Figure 2B**). Although the truncated MSLN variant expressed at comparable levels relative to the full-length construct (data not shown), the selected blocker was more sensitive to the truncated form (**Figure 2D**). Together, these data suggest that, in addition to the height of the B-antigen (T_B_), the location of the epitope (E_B_) may also influence blocking function (**Figures 1A**, **2A**). However, although targeting membrane-proximal epitopes shortens E_B_, it also leads to a proportionally larger X_B_ value that may sterically prevent the formation of the blocker complex.

### Section 3: controlling function with receptor hinge lengths

In the previous sections, we described how antigen height and epitope location may influence formation of the blocker complex and subsequent blocking function. Although targeting membrane-proximal epitopes of small antigens is a valid strategy for antigen selection, this is not always feasible from a therapeutic standpoint. Therefore, we investigated the height of the blocker receptor itself (R_B_) and how this parameter affects function. This posed a challenge, as the hinges typically used for CAR design are flexible, complicating the estimation of R_B_ (**Supplementary 2**) (10, 32).

**Figure 3:**
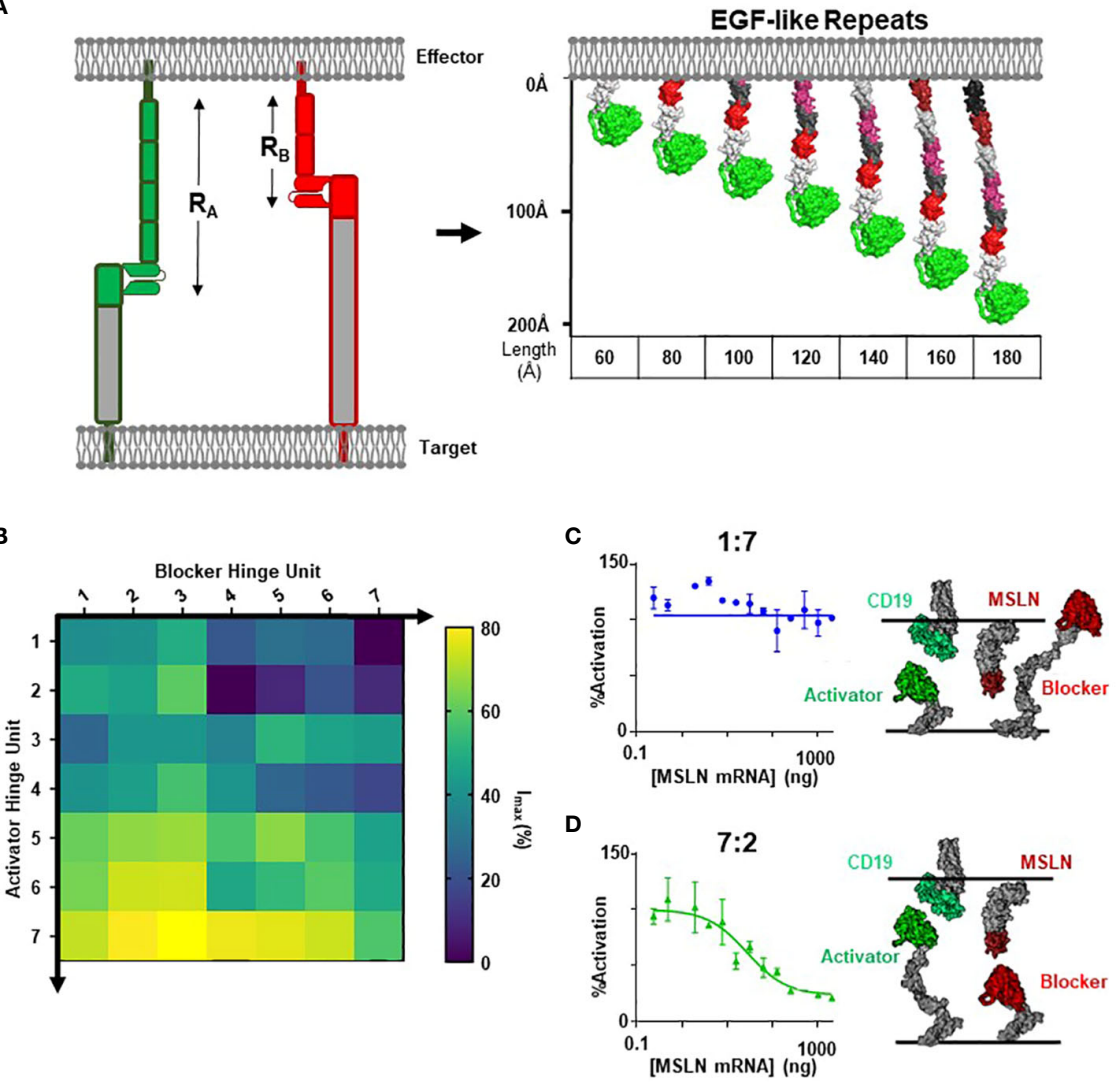
Modular Hinges Can Rescue a Membrane Distal MSLN Blocker. **(A)** Schematic of EGF-like hinges to manipulate receptor length. Extension from cell membrane estimated to be ~20-30Å per subunit. See **Table 1** for parameter definitions. **(B)** Jurkat T cells expressing each hinge combination of CD19 activator plus MSLN blocker were co-cultured with either K562(MSLN-) or K562(MSLN+) target cells. The difference in activation between the two conditions was converted into I_max_ (%) and presented as a heatmap. **(C)** Response of Jurkat T cell expressing 7EGF Activator: 2EGF Blocker in response to K562 target cells expressing increasing amounts of MSLN in co-culture assay (left), with schematic illustrating approximate activator and blocker interactions (right). **(D)** Response of Jurkat T cell expressing 1EGF Activator: 7EGF Blocker in response to K562 target cells expressing increasing amounts of MSLN in co-culture assay (left), with schematic illustrating approximate activator and blocker lengths (right).

To thoroughly define the relationship between receptor length (R_B_) and blocker function, we leveraged modular domain repeats that allow for estimation of the distance of the LBD from the T cell membrane surface based on the number of repeats (**Figure 3A**). Epidermal growth factor (EGF)-like domains, characterized by three intradomain disulfide bonds, were particularly attractive due to the high and uniform expression levels when incorporated as hinge subunits (**Supplementary 4**). Thus, we designed a series of blocker hinges that utilized up to 7 EGF-like domain repeats (extracted from LRP-1; uniprotID: Q07954, aa 4147–4409) to increase the distance between the MSLN ligand-binding domain and the T cell surface in 20-30Å increments (**Figure 3A**; **Table 2**). To characterize the geometric relationship between CAR and blocker, we also incorporated the same rigid hinge series into the CD19 CAR. Blocking efficiencies of each hinge combination were used to generate a 7x7 functional matrix, enabling a systematic evaluation of different activator/blocker geometries.

To compare blocking strength, we utilized a simplified inhibition assay in which we transfected each hinge combination into Jurkat cells and co-cultured these cells with either MSLN(+) or MSLN(-) K562 target cells engineered to overexpress CD19. We then calculated the percent decrease in activation signal associated with MSLN expression. Populating a matrix with these values revealed that short blocker hinges paired with long activator hinges led to significantly improved blocking, whereas short activators paired with long blockers completely abrogated blocker function (**Figure 3B**). Comparing two distinct hinge configurations in a MSLN titration co-culture yielded the full span of blocking profiles (**Figures 3C, D**). Furthermore, structural models suggest that the short CD19 activators sterically prevent the blocker from engaging the MSLN B-antigen (i.e., the blocker complex is too large; **Figure 3C**), whereas the hinge combination that best compensated for differences in the target antigens led to the most efficient blocking profile (**Figure 3D**).

**Figure 4:**
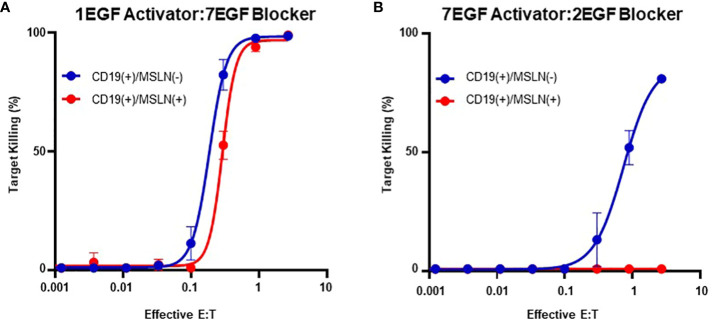
Modular Hinges Can Regulate Selectivity and Sensitivity. **(A)** PBMCs expressing a short CD19 activator and long MSLN blocker kill target cells expressing CD19 independently of MSLN expression status. **(B)** PBMCs expressing a long activator and short blocker require higher E:T ratios to kill target cells expressing CD19, but completely spare target cells expressing both CD19 and MSLN.

However, although blocking was most effective with long activators, extended activators were also associated with a decrease in activation sensitivity (**Supplementary 4**). This is consistent with reports highlighting that longer CAR hinges can negatively influence T cell activation (14), and these data suggest that the preference for short activator hinges imposes a constraint on blocker function. To confirm this observation in primary T cells, we transduced PBMCs with the two Tmod constructs presented in **Figures 3C and D**. Consistent with our observations in the Jurkat inhibition assay, PBMCs transduced with the short-hinged activator and long-hinged blocker responded strongly to CD19(+)MSLN(-) target cells, but also killed CD19(+)MSLN(+) target cells – indicating a failure to protect cells expressing the MSLN blocker antigen (**Figure 4A**). In contrast, PBMCs expressing long-hinged activators and short-hinged blockers were less effective at killing the CD19-only target cells, but completely spared the cells expressing both CD19 and MSLN (**Figure 4B**). Together, these data suggest that one approach to achieve compatibility with a suboptimal Tmod antigen pair is to modulate receptor lengths, leveraging a trade-off between selectivity and sensitivity.

In summary, the model CD19/MSLN target system explored above suggests that several geometric parameters (length of the receptor, antigen, and overall complex) are directly related to T cell function. These predictors contextualize the data for the Tmod antigen pairs examined in section 1 (CD19/HLA-A*02, CD19/MSLN, CD19/ICAM-1), and offer a rational explanation for the superior inhibitory performance of smaller B-antigens. Furthermore, the observations suggest a set of design principles to predict and improve blocker performance: (i) the blocker complex should be less than or equal to the activator complex and (ii): the B-antigen epitope must be accessible to the blocker on the cell surface, following engagement of A-Antigen by the activating CAR (**Supplementary 6**).

### Section 4: prospective test of geometric parameters for Tmod design: application to other targets

**Figure 5:**
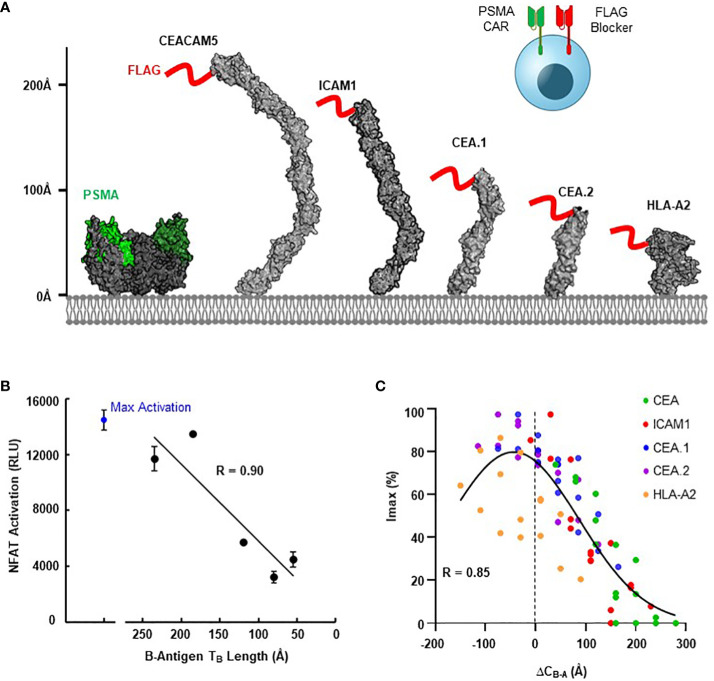
Blocking Function is Dependent on Antigen Context. **(A)** Structural comparison of PSMA A-antigen (with proposed epitope in green (35)) and a series of B-antigens with an N-terminal FLAG tag epitope (red), with axial length approximations for each antigen. **(B)** Comparison of NFAT activation signal from Jurkat T cell co-culture assays for each blocker antigen as a function of axial length (T_B_), using CD8/LIR-1 hinge configuration. **(C)** Correlation between I_max_ and the estimated difference in complex length between activator and blocker (ΔC_B-A_) using modular EGF hinges.

We next explored the generalizability of these design principles and engineering strategies when targeting a more extreme height disparity with different activator and blocker antigens. For the A-antigen, we selected the well-documented tumor associated antigen PSMA, for which many CARs targeting the membrane-distal region are available (33, 34). For the B-antigens, we considered HLA-A*02, ICAM-1, and a series of CEACAM5 variants to fully diversify the B-antigen axial length. To control variability between distinct blocker LBDs due to differences in expression, affinity, and epitope accessibility, we developed a blocker directed towards an engineered FLAG tag present on the N-terminus of each B-antigen target (**Figure 5A**) (36). Thus, any differences observed in Jurkat activation should be a direct result of differences in the target antigen construct.

Consistent with our observations presented in **Figure 1C**, the ability of the FLAG-blocker to inhibit the PSMA activation signal was closely correlated with B-antigen axial length (R=0.9; **Figure 5B**). Jurkat cells expressing the PSMA activator and FLAG-directed blocker only inhibited ~30% of the activation signal when co-cultured with PSMA(+)/FLAG-CEA(+) HeLa target cells (relative to co-culture with PSMA(+)/FLAG(-) target cells). In contrast, the same Jurkat cells inhibited over 80% of the maximum activation signal when co-cultured with target cells expressing the smallest FLAG-tagged B-antigens (**Figure 5B**). Differences in B-antigen expression did not explain these differences in function, as each B-antigen expressed at similar, high levels (>500,000 antigen-binding capacity (ABC) quantified by QIFI) (**Supplementary 5A**).

Leveraging this diverse functional behavior in response to antigen height, we then attempted to optimize blocking function for each FLAG-tagged B-antigen with the modular EGF hinges described in section 3. We incorporated a series of modular hinges into both the PSMA activator and FLAG blocker to generate a 4x4 matrix of Jurkat cells expressing both receptors. The Jurkat cells expressing long activators and short blockers were generally more capable of inhibiting the maximum activation signal when co-cultured with PSMA(+)/FLAG(+) HeLa target cell lines, regardless of B-antigen length (**Supplementary 5B**). Indeed, the hinge extensions improved Tmod function with even the largest FLAG-CEA and FLAG-ICAM-1 B-antigens, although the resultant long activator hinge reduced the baseline signal.

To compare these datasets in a single analytical framework we estimated the length of each receptor-ligand complex using a conversion that sums the axial length of the receptor (**Table 2**) with the axial length of the target antigen (**Table 3**). After calculating the maximum percentage of inhibition (I_max_), we plotted the measured values against the difference in estimated length between the activation and blocking complexes (ΔC_B-A_, **Supplementary 5C**). Despite the cumulative inaccuracies associated with estimating the length of both receptor and antigen, the data followed a general nonlinear trend with peak inhibition occurring when ΔC_B-A_ approaches zero. Plotting all the data together revealed an approximate Gaussian distribution, even with limited data points on the lefthand (i.e., activator smaller than blocker) side (**Figure 5C**). Thus, any gross mismatch between activator and blocker complex has the potential to limit Tmod function, but rational receptor design can correct for suboptimal conditions.

**Table 2:** Estimation of receptor lengths with increasing hinge subunits.

EGF-like Hinge unit	R Estimate (Å)
1	60
2	80
3	100
4	120
5	140
6	160
7	180

**Table 3:** Estimation of antigen pair sizes.

Target Combinations					
A-Antigen	B-Antigen	TA	TB	EA	EB
CD19	MSLN	45	90	45	90
PSMA	CEA	75	235	75	235
PSMA	CEA.1	75	120	75	120
PSMA	CEA.2	75	80	75	80

## Discussion

Tumor *vs*. normal selectivity is one of the key challenges to the development of improved cancer medicines. Consequently, new targets and targeting mechanisms are desperately needed. The Tmod approach offers the prospect of achieving selectivity by exploiting antigen loss or expression differences, potentially providing access to a new set of target antigens (4). Tmod leverages NOT-gated CAR-T cells to target tumor-specific antigen profiles rather than tumor-associated single antigens. However, the introduction of a second receptor as a blocker module creates new obstacles for the development of cell therapies. In addition to the complexities associated with design and co-expression of two receptors, targeting pairs of diverse antigens poses unique challenges.

These challenges involve not only the details of tissue-specific gene expression but also the varied structural features of surface antigens. With regard to requirements for Tmod function, the most relevant structural difference may be the disparate axial dimensions of extracellular domains. Though Tmod modularity has been repeatedly demonstrated in the context of HLA-directed blockers, it has not been explored in detail in a broader context. We hypothesized that the original blocker hinge, developed for HLA antigens, may be biased toward shorter proteins. To probe the mechanism of Tmod function, we created a platform where the activator and blocker receptors could be systematically tuned to promote the formation of optimal synapses, depending on the relative height and epitope locations of the target antigens. We replicated observations from the CAR field and showed that extending the LBD of the CAR with a modular hinge reduced T cell activation (13, 14), yet also demonstrated that this extension may be required for targeting larger antigens in the context of Tmod. In these cases where the blocker antigen is significantly larger than the activator, the reduction in activation sensitivity can be offset by a significant gain in selectivity. Thus, a Tmod construct solution determined for one antigen pair may not be the optimal solution for other combinations due to axial length differences that can dramatically influence function. To help design the Tmod construct appropriate for specific antigen pairs, we defined a simple predictive model that estimates blocker function based on the relative height disparity between the blocker and activator.

The set of design principles delineated here provides confidence that many antigen combinations can be addressed with the Tmod platform, beyond those that involve HLA antigens. These results are important given the limitations not only imposed by HLA LOH frequencies in cancer but also the finite number and the nature of gene products encoded by the human genome. In this regard, it may not always be feasible to choose antigens with optimal properties. In situations where other possibilities have been exhausted (e.g., choice of antigens based on tissue expression, axial length, and LBD affinity which has been shown to play a substantial role in CAR sensitivity (37)), these data would suggest that in most cases Tmod selectivity can be improved by adjusting the length of the activator and blocking receptors. Furthermore, although our findings were directly applied to the Tmod platform, the relationship with activating CARs will likely translate to other logic-gated strategies to regulate CAR activity (6).

The relationships between epitope location, antigen height, and signal transduction outlined here are reminiscent of other models for T cell activation (e.g., the kinetic segregation model) which incorporate the fact that extracellular domains of activating proteins (TCRs, CD28, etc.) are smaller than inhibitory components (e.g., CD45 and CD148 (16, 38)). Multiple groups have demonstrated that activating CAR function is also dependent on target size and epitope location. In some cases, increasing the extracellular length of the CAR is sufficient to disrupt T cell activation (14). Our data demonstrate that a similar set of principles applies to the more complex phenomenon of antigen-dependent inhibition, consistent with an elaborate organization of receptors in the CAR-T synapse (39). Indeed, our findings suggest the blocker must colocalize with engaged activating CARs to exert their inhibitory effect, presumably by recruiting phosphatases to reverse the activator-driven phosphorylation (40). This would suggest the Tmod platform integrates signals similarly to NK cells, where matching receptor/ligand sizes has also been demonstrated to be crucial for cell function (41). However, the geometric relationships observed here are just one of the multiple factors that control T cell function. Other variables, such as antigen density, target affinity, and receptor expression levels also affect function. Additionally, the Jurkat assay we used to screen different combinations is an artificial system that simplifies many of the complexities of the immune system. As such, targeting specific epitopes or manipulating hinge lengths will likely have little to no effect on unrelated therapeutic criteria, such as T cell persistence and fitness (42).

In summary, our data provides a general framework for optimizing logic-gated T cell therapeutics with focus on antigen selection and receptor design. Further studies will help connect our *in vitro* observations to *in vivo* function and ultimately to the clinic. In addition, despite the utility of using structural models and simple geometric parameters to help predict optimal Tmod designs, many details of Tmod function remain to be elucidated (e.g., the detailed nature of the interactions between the activator and blocker). The findings presented here, which demonstrate a clear relationship between Tmod receptor length and function, are a step on the path toward a more complete understanding of engineered dual-receptors, a potentially significant advance for cancer cell therapy.

## Data availability statement

The raw data supporting the conclusions of this article will be made available by the authors, without undue reservation.

## Ethics statement

Ethical approval was not required for the studies on humans in accordance with the local legislation and institutional requirements because only commercially available established cell lines were used.

## Author contributions

AP: Writing – original draft, Data curation, Formal Analysis, Investigation, Writing – review & editing. RB: Data curation, Formal Analysis, Investigation, Writing – original draft. SS: Investigation, Writing – review & editing, Methodology. EV: Investigation, Writing – review & editing, Formal Analysis. AW: Methodology, Writing – review & editing. MD: Methodology, Writing – review & editing. CG: Writing – review & editing. CJ: Writing – review & editing, Conceptualization. BD: Writing – review & editing. MS: Supervision, Writing – review & editing. AH: Supervision, Writing – review & editing. AK: Supervision, Writing – review & editing, Resources. TR: Conceptualization, Supervision, Writing – original draft, Writing – review & editing.
