# Supplementary material for: Geometric parameters that affect the behavior of logic-gated CAR T cells
Source: Front Immunol. 2024 Jan 26;15:1304765. doi: 10.3389/fimmu.2024.1304765 (PMC10853413; doi:10.3389/fimmu.2024.1304765)
Supplement: Supplementary file 1 [file DataSheet_1.pdf]

Supplement 1

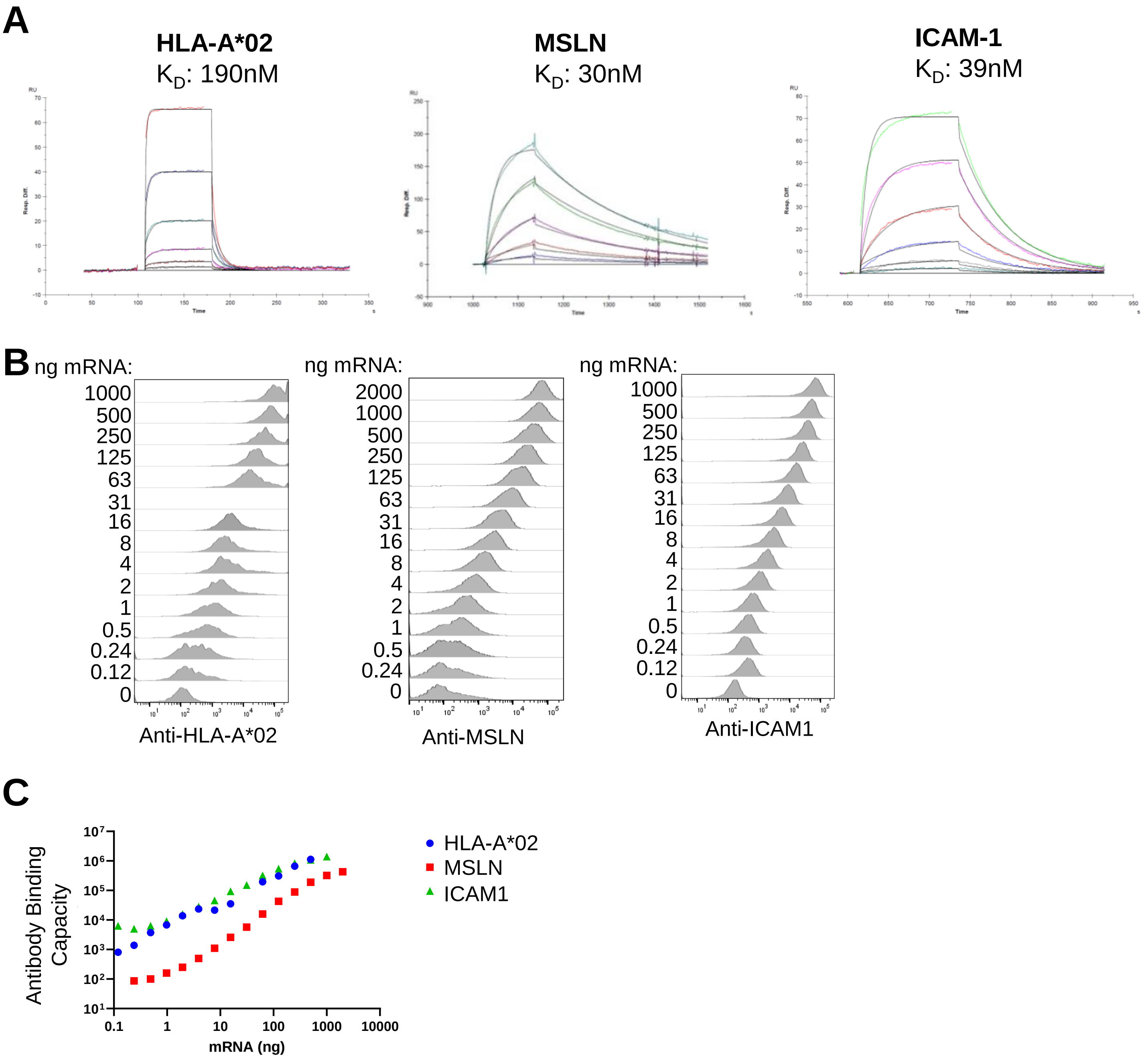

(A) scFv affinities for B-antigens shown in Figure 1. (B) mRNA titration of B-antigens on target cells. (C) Relative expression levels quantified by QIFI.

Supplement 2

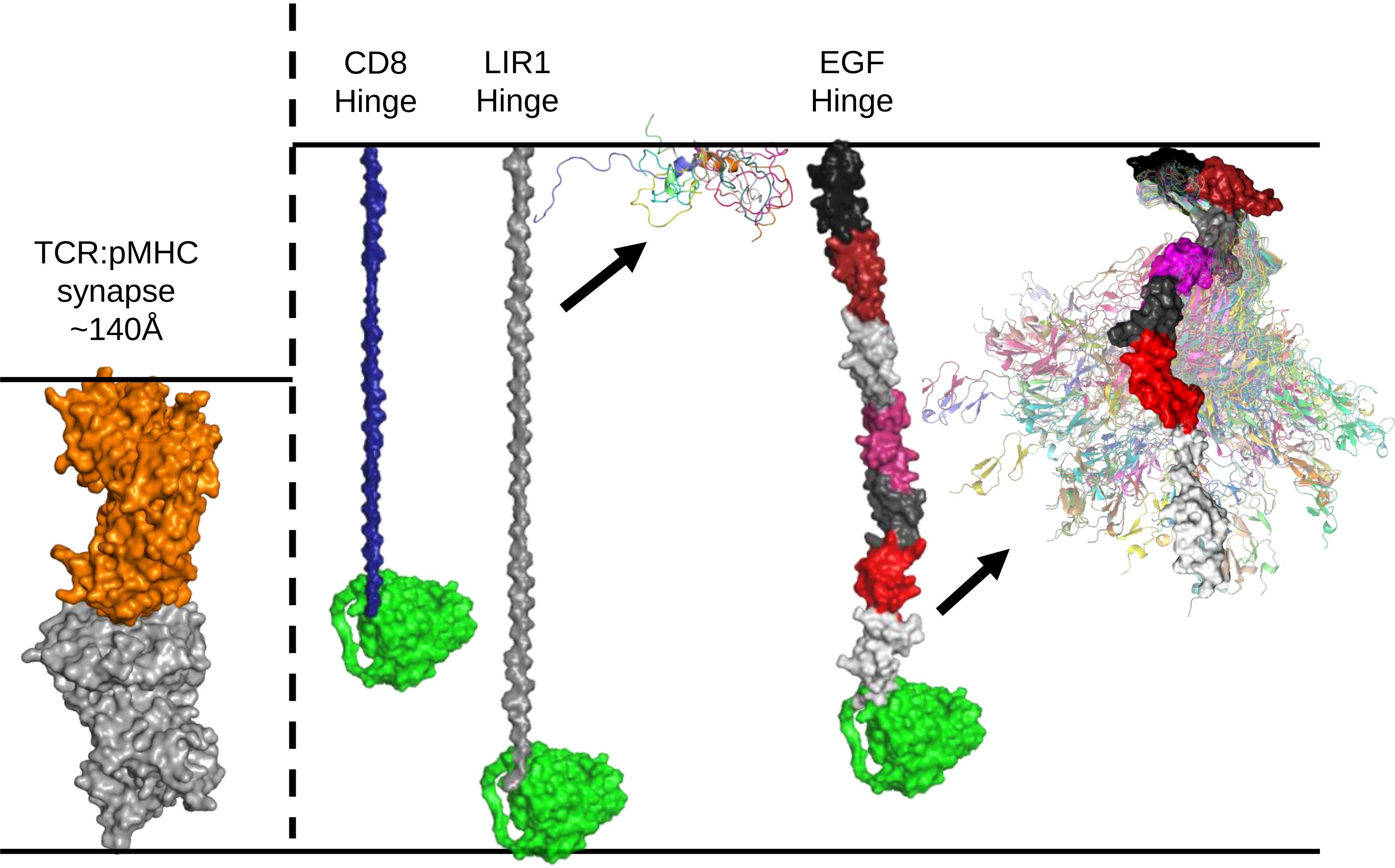

Comparison of Hinge Length and Flexibility relative to TCR:pMHC complex. 100 Monte Carlo simulations for each hinge were overlayed to assess flexibility.

Supplement 3

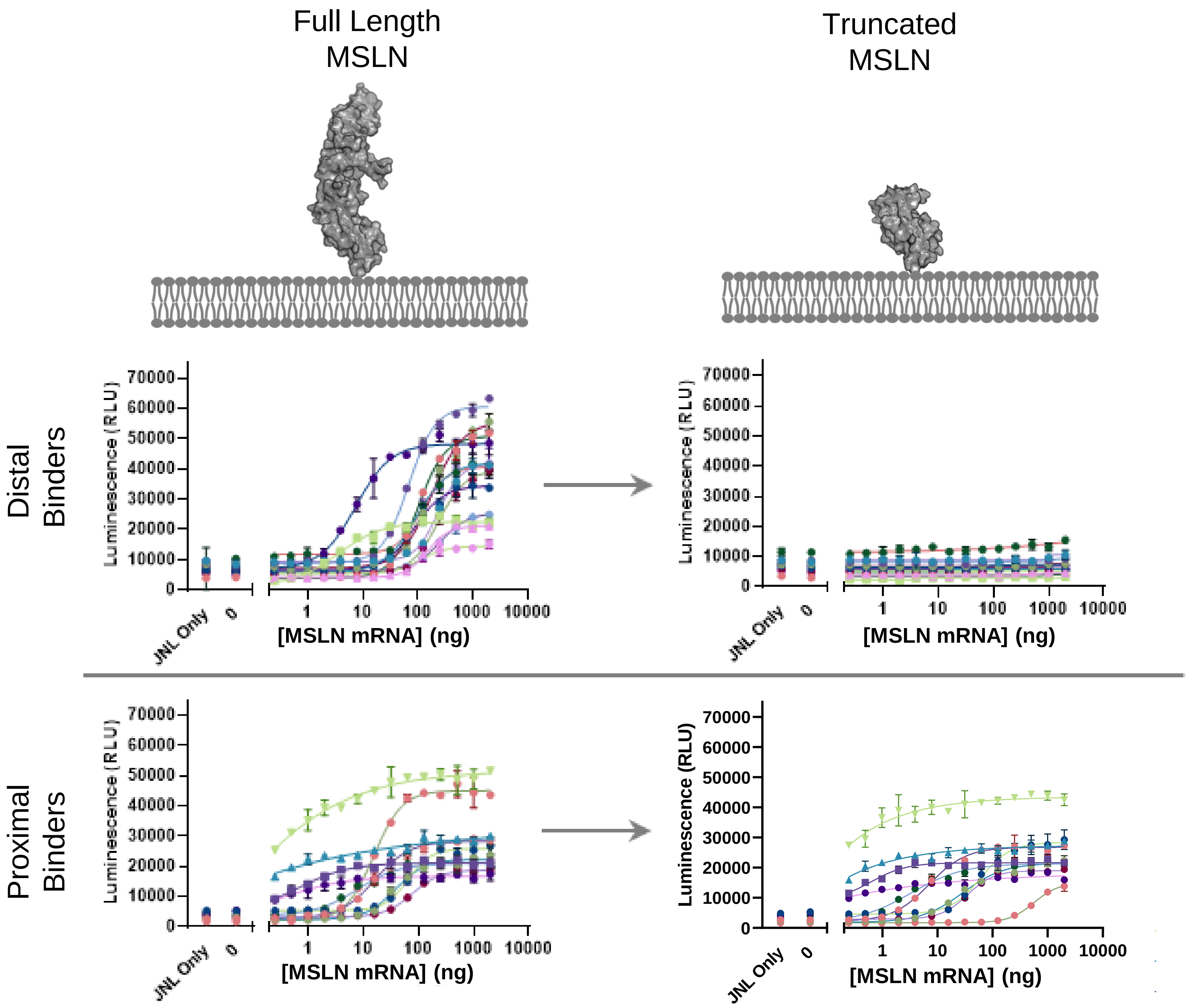

Functional assessment of MSLN CARs responding to full length or truncated MSLN. If a CAR did not respond to truncated form, it was classified as a distal binder

Supplement 4

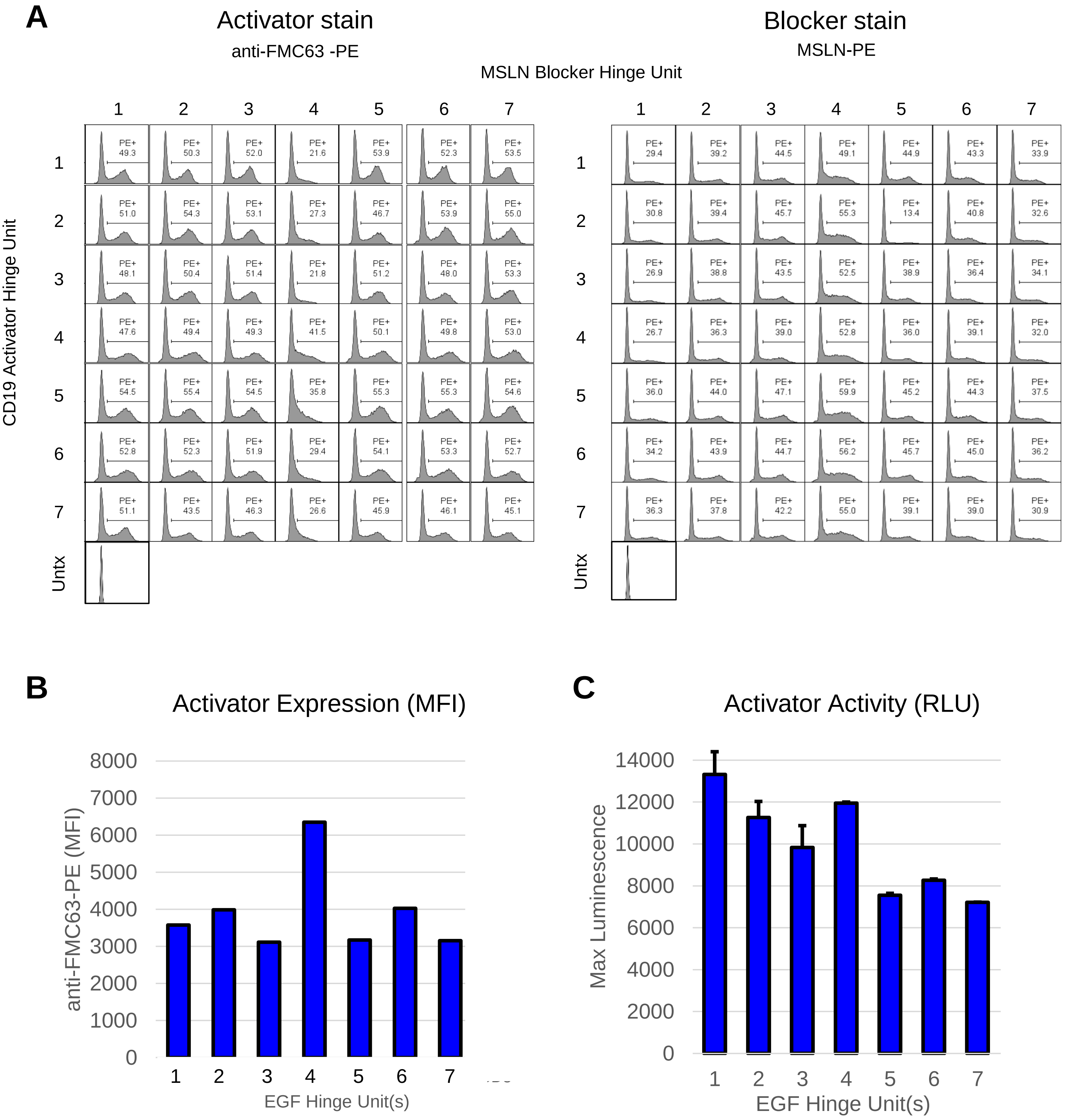

(A) Expression levels CD19 activator/MSLN blocker with EGF hinges. Untx, untransfected. (B) Relative expression levels of activator. (C) Max activation decreases as hinge length decreases independent of expression.

Supplement 5

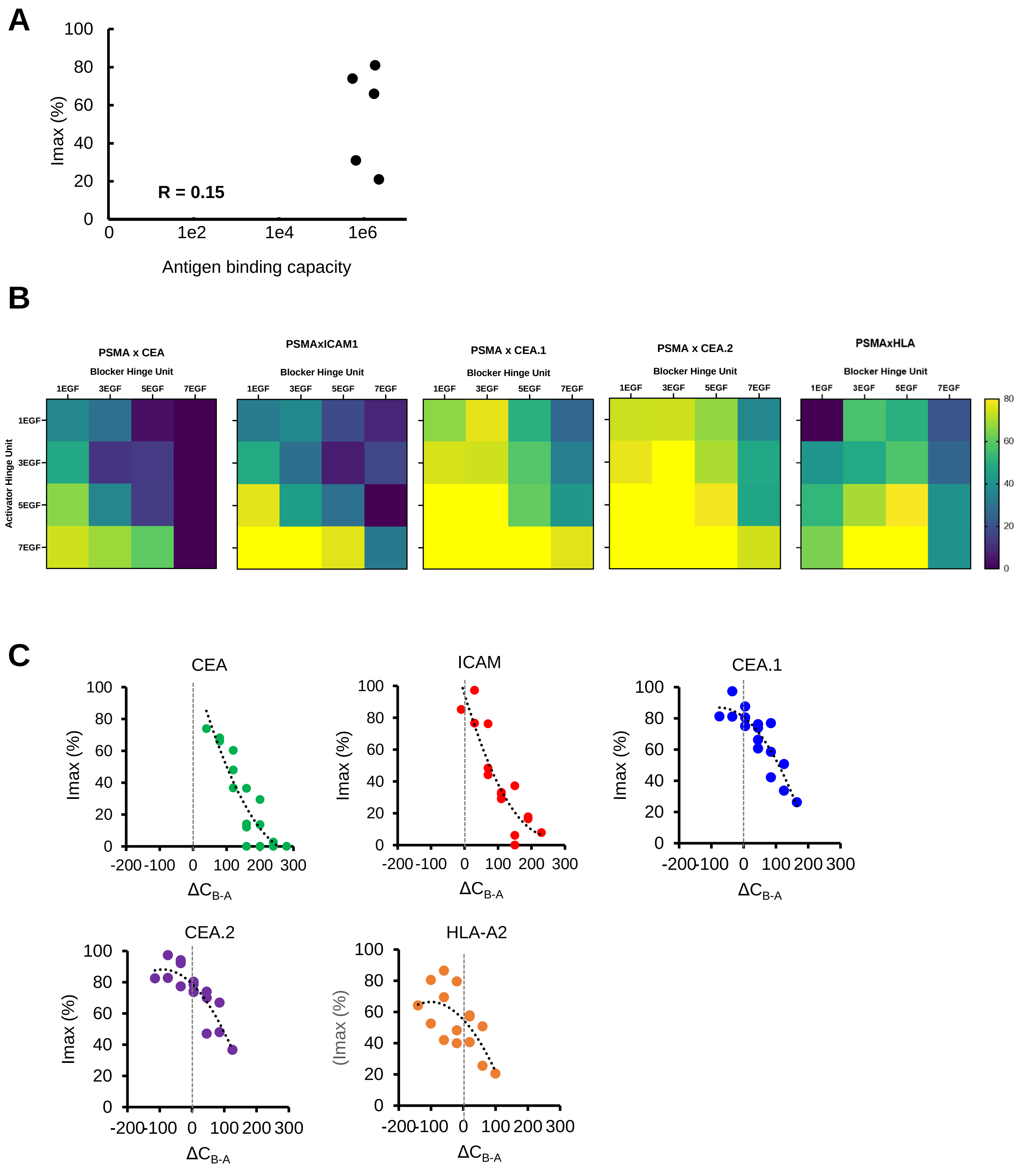

(A) Antigen density for FLAG-antigens exceeds 500k ABC and is uncorrelated with Imax. (B) Blocking profile when expanding the R_A or R_B with EGF modular hinges. (C) Plotting the Imax data from (B) versus total estimates of ΔC.

Supplement 6

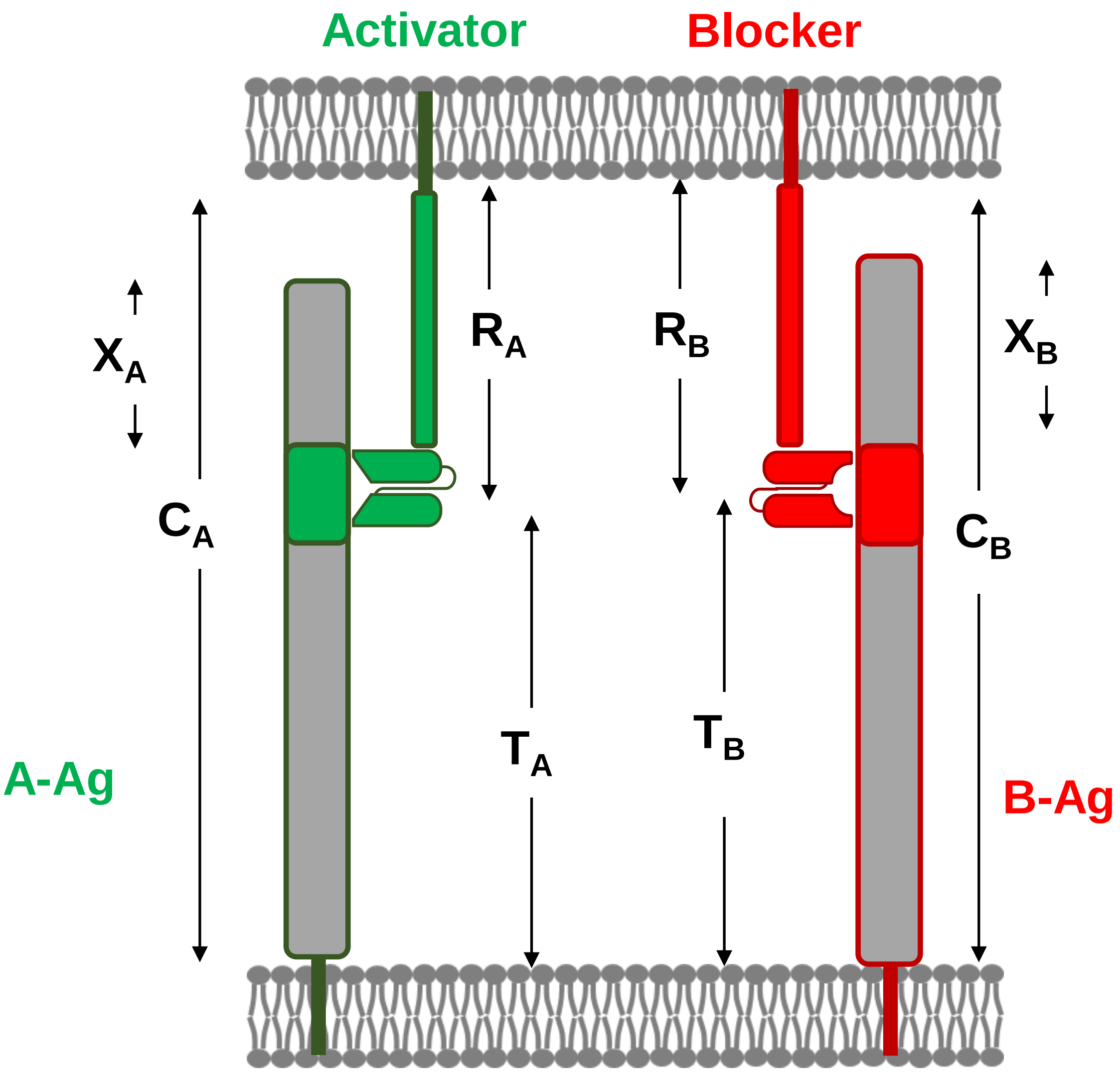

Full diagram of geometric parameters.
